# A multimodal neuroimaging classifier for alcohol dependence

**DOI:** 10.1038/s41598-019-56923-9

**Published:** 2020-01-15

**Authors:** Matthias Guggenmos, Katharina Schmack, Ilya M. Veer, Tristram Lett, Maria Sekutowicz, Miriam Sebold, Maria Garbusow, Christian Sommer, Hans-Ulrich Wittchen, Ulrich S. Zimmermann, Michael N. Smolka, Henrik Walter, Andreas Heinz, Philipp Sterzer

**Affiliations:** 1 Department of Psychiatry and Psychotherapy, Charité – Universitätsmedizin Berlin, corporate member of Freie Universität Berlin, Humboldt-Universität zu Berlin, and Berlin Institute of Health, Berlin, Germany; 2 Department of Psychiatry and Psychotherapy, Technische Universität Dresden, Dresden, Germany; 3 Neuroimaging Center, Technische Universität Dresden, Dresden, Germany; 4 Institute of Clinical Psychology and Psychotherapy, Technische Universität Dresden, Dresden, Germany; 5 Department of Psychiatry and Psychotherapy, Ludwig Maximilans Universität Munich, Munich, Germany

**Keywords:** Addiction, Diagnostic markers

## Abstract

With progress in magnetic resonance imaging technology and a broader dissemination of state-of-the-art imaging facilities, the acquisition of multiple neuroimaging modalities is becoming increasingly feasible. One particular hope associated with multimodal neuroimaging is the development of reliable data-driven diagnostic classifiers for psychiatric disorders, yet previous studies have often failed to find a benefit of combining multiple modalities. As a psychiatric disorder with established neurobiological effects at several levels of description, alcohol dependence is particularly well-suited for multimodal classification. To this aim, we developed a multimodal classification scheme and applied it to a rich neuroimaging battery (structural, functional task-based and functional resting-state data) collected in a matched sample of alcohol-dependent patients (N = 119) and controls (N = 97). We found that our classification scheme yielded 79.3% diagnostic accuracy, which outperformed the strongest individual modality – grey-matter density – by 2.7%. We found that this moderate benefit of multimodal classification depended on a number of critical design choices: a procedure to select optimal modality-specific classifiers, a fine-grained ensemble prediction based on cross-modal weight matrices and continuous classifier decision values. We conclude that the combination of multiple neuroimaging modalities is able to moderately improve the accuracy of machine-learning-based diagnostic classification in alcohol dependence.

## Introduction

In recent years, technical advancements in magnetic resonance imaging (MRI) technology and increasing access to these state-of-the-art MRI facilities for both clinicians and researchers have nourished the quest for MRI-based diagnostic classifiers of psychiatric disorders that proceed in an automated and objective manner. In addition, multiple MRI modalities, including high-resolution structural images, resting-state connectivity maps, white matter tractography based on diffusion tensor imaging and functional MRI, are now readily available as part of standard experimental protocols. The hope associated with such *multimodal MRI* batteries is that measurements targeting different levels of brain structure and function will, in combination, lead to a breakthrough in the quantitative characterization of psychiatric disorders^1–3^.

A limiting factor in this endeavour is that most psychiatric disorders have an upper bound for the accuracy of machine-learning-based diagnostic classification imposed by (1) ‘label noise’ of psychiatric diagnoses, evidenced by often poor inter-rater reliabilities^4,5^, and (2) an intrinsic heterogeneity of psychiatric diagnostic labels themselves^6–8^. It is therefore no surprise that the most successful applications of (multimodal) MRI-based machine learning have been achieved in disorders involving clear neurodegenerative effects such as Alzheimer’s disease^9–12^ or multiple sclerosis^13,14^.

Here, we reasoned that alcohol dependence is a well-suited *psychiatric* disorder for automated diagnostic classification based on multimodal MRI and an ideal test case and benchmark for methodological developments. This is first and foremost because neurobiological correlates of alcohol dependence have been established at several levels of description, including grey-matter loss^15–18^, increased ventricular size/cerebrospinal fluid concentration^19–21^ and aberrant neural reward responses^22–25^. The diversity of effects in combination with a high degree of replicability could make the combination of different MRI modalities particularly powerful in the case of alcohol dependence. In addition, alcohol dependence, relative to other psychiatric disorders, is a reliable diagnosis^26–28^ and thus a paradigmatic case to gauge the true predictive potential of an MRI-based classifier for psychiatric diagnosis.

Our investigation was based on a sample of 119 alcohol-dependent patients and 97 controls who underwent an extensive neuroimaging battery including structural^16,29^, functional^30–32^ and resting-state^33^ MRI as part of the LeAD study (www.lead-studie.de; clinical trial number: NCT01679145). Our guiding rationale for the construction of a multimodal diagnostic classifier was clinical utility and practicability, and thus to leverage on neuroimaging modalities that are effective and simple to acquire (i.e., relatively short acquisition times and standard scanning sequences) and to analyse (e.g. no computational cognitive modelling or other approaches rich on assumptions). Selected modalities comprised grey-matter density, cerebral spinal fluid and cortical thickness based on structural MRI as well as basic reward responses and resting-state connectivity based on functional MRI.

Our goal was to address key methodological challenges of multimodal MRI, such as the heterogeneity of different modalities in terms of statistical properties and the number of predictors, missing data in one or more modalities, and the relative weighting of each modality. In the present investigation we thus developed a novel classification framework that (1) combined modality-specific predictions in an ensemble vote, (2) allowed for emphasizing or de-emphasizing individual modalities through weighting, (3) considered fine-grained information from modality-specific classifiers instead of binary labels, and (4) was robust to missing data in individual modalities. We hypothesized that the combination of MRI modalities for diagnostic classification of alcohol dependence would outperform any individual unimodal classification approach.

## Method

### Participants

This study was conducted as part of the Learning and Alcohol Dependence (LeAD) study, a German (Berlin, Dresden) program investigating the neurobiological basis of alcohol dependence (www.lead-studie.de; clinical trial number: NCT01679145^30,34,35^). We assessed 119 individuals aged 20–65 (18 female) meeting criteria of alcohol dependence according to ICD-10 and DSM-IV-TR (American Psychiatric Association 2000) and 97 healthy controls aged 21–65 (16 female) matched in terms of age, gender and smoking (see Table 1).

**Table 1:** Sample characteristics for alcohol-dependent (AD) and healthy control (HC) subjects.

	AD (N = 119)			HC (N = 97)			t or 𝜒^2^	df	p
	Mean	SD	%	Mean	SD	%			
Gender [female]			15.1			16.5	0.008	N = 216	0.93
Age [years]	45.0	10.7		43.6	10.8		0.9	214	0.38
Education [years]	10.5	0.1		11.2	0.2		−3.4	207	<0.001
SES	−0.4	0.2		0.7	0.3		−3.6	170	<0.001
Smokers			76.5			67.0	1.9	N = 216	0.16
ADS score	14.8	6.9		2.0	3.0		17.0	213	<0.001
AD duration [years]	11.7	9.9						N = 110	
Amount life [kg]	1805	1121		286	811		11.1	214	<0.001
Amount past year [kg]	178	13		11	1		12.0	214	<0.001
OCDS total score	11.9	8.5		2.8	2.8		10.1	207	<0.001
BIS-15 total score	31.6	6.5		29.1	5.5		2.9	205	0.004
TMT (percentile)	36.1	25.1		44.8	25.1		2.5	209	0.014
DSST	64.3	15.1		73.5	16.6		4.2	211	<0.001
DSB	6.5	1.9		7.4	2.0		3.4	214	0.001
MWT	104.7	9.4		104.5	8.9		−0.2	209	0.82
Wordlist	90.8	16.1		90.9	14.1		−0.0	209	0.97

Socioeconomic status (SES): sum of z-transformed self-ratings of social status, household income and inverse personal debt scores^98^; Alcohol Dependence Scale (ADS): degree/level of AD^99^; Amount life: lifetime alcohol consumption in kilograms based on the CAPI-CIDI (Wittchen and Pfister, 2007; Jacobi *et al*., 2013); Amount past year: alcohol consumption during the past year in kilograms based on the CAPI-CIDI (Wittchen and Pfister, 2007; Jacobi *et al*., 2013); Obsessive Compulsive Drinking Scale (OCDS): Current craving for alcohol^100^; Barratt Impulsiveness scale (BIS-15): impulsivity^101^; Trail making test (TMT; percentile): visual attention and task switching^102^; Digit symbol substitution test (DSST): processing speed^103^. Digit span backwards (DSB): working memory span^103^. Multiple-choice vocabulary intelligence test (Mehrfachwahl-Wortschatz-Intelligenztest, MWT): crystallized / verbal intelligence^104^; Wordlist (savings): wordlist memory test^105^.

We used the computer-assisted interview version Composite International Diagnostic Interview (CAPI-CIDI^36,37^) to verify diagnosis criteria of AD in the patient group and to exclude the possibility of AD in control subjects. For inclusion, individuals with AD had to meet criteria for AD for at least three years and had to undergo an inpatient detoxification phase (average duration ± SEM: 22.8±1 days). Exclusion criteria for all subjects were left-handedness (Edinburgh handedness index below 50^38^), contraindications for MRI, and a history of or current neurological (including Korsakoff syndrome) or mental disorders (excluding nicotine dependence in both groups and alcohol abuse in individuals with AD, but including abuse of other drugs). Mental disorders were assessed according to DSM-IV axis one as verified by the CAPI-CIDI. It was ensured that all subjects were free of psychotropic medication (including detoxification treatment) known to interact with the central nervous system for at least four half-lives. Current non-alcohol drug abuse was confirmed by means of a dedicated urine test.

Note that several sampling characteristics were influenced by the fact that predictors of relapse, while not part of the present work, were another research focus within the LeAD study^30^: (1) to have sufficient power for the comparison between relapsers and abstainers, the AD group was oversampled relative to the HC group; (2) to be able to study relapse behaviour, detoxification at baseline was an inclusion criterion; (3) to prevent the possibility that handedness would be unequally distributed across relapsers and abstainers, only right handers were included. Finally note that matching for smoking implied a higher proportion of smokers in the HC sample than expected from a representative sample (67% versus a representative prevalence of 28% in Germany^39^).

Participants gave written informed consent. Ethical approval for the study was obtained from both sites (Ethics committee of the Universitätsklinikum Dresden/Technische Universität Dresden, EK 228072012; Ethics committee of Charité–Universitätsmedizin Berlin, EA 1/157/11), and procedures were in accordance with the Declaration of Helsinki.

### Overview of neuroimaging modalities

To maximize the clinical utility of a multimodal diagnostic classifier, we constructed a neuroimaging battery of five modalities that was (1) simple to acquire and to analyse, and (2) based on established effects either in previous literature or on own works within the LeAD program. From structural MRI measurements we derived three modalities: (1) grey-matter density, as damaging effects to grey-matter integrity in chronic alcoholics have been replicated numerous times in the literature^15,17,18^ including our own work in which we also demonstrated high diagnostic discriminability^16^; (2) cerebrospinal fluid, as increased ventricular size (or increased cerebrospinal fluid, respectively) is arguably one the most salient characteristics of alcoholic brains^19–21^; and (3) cortical thickness, an additional specific marker of grey-matter integrity obtained through surface-based analysis, which has been successfully used in more recent studies to characterize structural damage in alcohol dependence^40–42^.

From task-based functional MRI we obtained (4) functional activation patterns representing a basic reward response (outcome versus no outcome), motivated by the fact that aberrant functional reward responses have been consistently found for alcohol dependence^22–25^. Finally, from resting-state functional MRI, we derived (5) nucleus accumbens whole-brain connectivity maps, as one of the key research goals of the LeAD program was testing a hypothesis about disturbed striato-frontal connectivity underlying the development of problematic drinking behaviours (this hypothesis has recently been confirmed by authors of this article^33^). Note that all data were linearly corrected for variance of no interest related to demographic variables age, gender and site.

### MRI data acquisition

Magnetic resonance imaging (MRI) was performed on a 3-Tesla Siemens Trio (Erlangen, Germany) scanner with a 12-channel head-coil to obtain (1) structural, (2) functional task-based and (3) functional resting-state MRI data. Structural T_1_-weighted MRI scans were acquired using a magnetization-prepared rapid gradient echo sequence (repetition time: 1900 ms; echo time: 5.25 ms; flip angle: 9°; field of view: 256 × 256 mm2; voxel size: 1 mm isotropic; 192 sagittal slices). Functional (task-based or resting-state) T_2_*-weighted MRI scans were acquired using a gradient echo planar imaging sequence (repetition time: 2410 ms; echo time: 25 ms; flip angle: 80°; field of view: 192 × 192 mm2; voxel size: 3 × 3 × 2 mm^3^) comprising 42 slices approximately −25° to the bicommissural plane. Volume-to-volume movement of more than 3 mm translation and/or 2 degrees rotation led to exclusion (9 HC and 12 AD subjects were excluded due to these criteria). All imaging data were screened for corrupted data or serious acquisition artefacts.

### MRI preprocessing and feature preparation

Neuroimaging features from overall five modalities were computed: grey-matter density (GMD), cerebrospinal fluid (CSF) and cortical thickness (CTH) from structural MRI; a basic reward response (RWR) signal from task-based functional MRI and nucleus accumbens connectivity (NAC) from resting-state functional MRI. In the following we describe the steps involved in preprocessing and feature preparation for each modality.

### Structural MRI

Structural MRI images were processed in two separate analyses streams for GMD/CSF and CTH. For GMD/CSF, SPM12 (http://www.fil.ion.ucl.ac.uk/spm) was used in combination with voxel-based morphometry (VBM8; http://dbm.neuro.uni-jena.de/vbm). T1-weighted Images were spatially normalized to a Montreal Neurological Institute (MNI) template and segmented based on tissue types. Unmodulated images representing GMD and CSF density were smoothed with an 8 mm isotropic Gaussian kernel. To reduce dimensionality, 110 regional averages for both GMD and CSF were computed based on a combined cortical and subcortical anatomical brain atlas^16,43^.

To obtain estimates of CTH, cortical reconstruction was performed on T1-weighted images using the FreeSurfer morphometric analysis suite (http://surfer.nmr.mgh.harvard.edu/). The technical details of these procedures are described in prior publications^44–55^. CTH estimates were obtained by calculating the closest distance from the grey/white boundary to the grey/CSF boundary at each vertex on the tessellated surface^45^. Average cortical thickness estimates were obtained for 358 brain regions based on the Glasser anatomical atlas^56^.

### Task-based functional MRI

Preprocessing was performed using SPM8 (www.fil.ion.ucl.ac.uk/spm) and included slice time correction, realignment to the first image, coregistration with the structural image, spatial normalization into MNI space and smoothing (8 mm Gaussian kernel). Details of the paradigm and the statistical first-level model have been provided elsewhere^30^. In brief, the paradigm involved a probabilistic value-based decision making task^57^ in which participants could receive a fixed monetary reward in each of 201 trials. While the goal of the paradigm is to distinguish model-based from model-free learning signals by means of a computational model, following our rationale of constructing a neuroimaging battery with only the most fundamental modalities, we here used the basic contrast of reward (+20 Euro cents) versus no reward.

Within the statistical first-level model^30^, we used the two onset regressors for *reward* and *no-reward* conditions during the outcome phase and computed directional contrasts (*reward* > *no reward)*. To reduce the extremely high dimensionality of individual fMRI whole-brain contrast maps (>200.000 valid voxels), we downsampled the data to 10 mm isotropic voxels resulting in 1461 valid voxels (features) for each participant. Exploratory classification analyses with smaller voxels sizes (2–8 mm in steps of 2 mm) showed that no relevant information was lost at 10 mm.

### Resting-state functional MRI

Preprocessing of resting-state data was performed using FSL (https://www.fmrib.ox.ac.uk/fsl) and included motion correction, slice timing correction, non-brain removal, 6 mm FWHM spatial smoothing. Data were denoised using independent component analysis (ICA-AROMA^58,59^), high-pass-filtered at 0.008 Hz and normalized to MNI space. To estimate whole-brain accumbens connectivity maps, binary seed masks of the left and right nucleus accumbens were defined using the Harvard-Oxford Subcortical Probability Atlas (50% probability threshold). The first Eigen time series was extracted from the preprocessed resting-state data, separately for the left and right nucleus accumbens. Finally, these time series were regressed against every other voxel’s time series using deep white matter and CSF as nuisance variables. To reduce dimensionality, we computed regional averages for the 110 anatomical regions of the JHU brain atlas^43^ and averaged across the left and right nucleus accumbens. Note that while participants were excluded when falling asleep during scanning, part of the interindividual variance in resting state connectivity may be explained by unknown interindividual differences in drowsiness/vigilance, which were not assessed in an objective manner in the current study. Nevertheless, only a small number of participants (7 AUD and 6 HC) reported brief moments of drowsiness or loss of vigilance during the exit interview.

### Unimodal diagnostic classification

In a first step, the diagnostic accuracy of each of the five modalities was assessed. Before submitting the modalities to classification, each modality was soft-normalized by removing the median and scaling the data according to a quantile range (1% to 99%)^60^. We considered two different classifier types: support vector machine (SVM^61^) and weighted robust distance (WeiRD^16,62,63^). SVM is arguably the most popular classifier in the context of neuroimaging^64^, as it robustly handles data with high dimensionality but few samples per class. We used the implementation provided by libsvm^65^ with a radial basis function kernel. WeiRD is a distance-to-centroid classifier (code available at https://github.com/m-guggenmos/weird), which assigns class labels based on the Manhattan distance to class prototypes in a statistically weighted feature space. A key advantage of WeiRD is the fact that it is parameter-free and hence does not require expensive optimization schemes. In previous work we have shown that WeiRD, despite its simplicity, performs surprisingly well across a range of neuroimaging classification problems^16,62,63^.

Both classifiers operated in a leave-one-out (LOO) cross-validation procedure, such that in each cross-validation fold, the classifiers were trained on the data of all but one participant and tested on the left-out participant. Each participant was left out and predicted exactly once. LOO was chosen over other cross-validation schemes, because it is approximately unbiased^66^, deterministic and computationally inexpensive. For SVM specifically, we additionally performed nested cross-validation and grid search to optimize the cost parameter *C* (range of *C*: 2^*x*^, *x* = −5:1:10). We used the balanced accuracy as a scoring metric and computed p-values of the null hypothesis that the balanced accuracy is equal to chance level^67^.

Note that the number of features outnumbered the number of samples. This is the standard scenario in multivariate neuroimaging analyses, which have been widely used with success to fit machine learning models within and across individuals^68,69^, most likely due to intercorrelations between features^70^.

In addition, we assessed the importance of each region for classification. For clarity and brevity, we show importance scores only for the superior classifier (SVM or WeiRD) of a modality. Where SVM was superior, we derived importance scores from the hyperplane-defining weight vector in feature space. Although the feature space is not the input space in the case of the radial-basis function kernel used here, the weights in feature space nevertheless provided a useful estimate of feature importance for descriptive purposes. Where WeiRD was superior, we used WeiRD votes as a measure of feature importance, as described previously^16^.

### Multimodal diagnostic classification

The aim of multimodal neuroimaging classification is to improve overall performance by leveraging on the combined information of more than one neuroimaging modality. Here we combined all five modalities introduced above (GMD, CSF, CTH, RWR, NAC) in an integrated classification scheme. The key design principle for the multimodal classification scheme was that a separate classifier was trained on each modality, which made it possible to select the optimal classifier for each modality and to fit it to the specific statistical properties of a modality.

**Figure 1:**
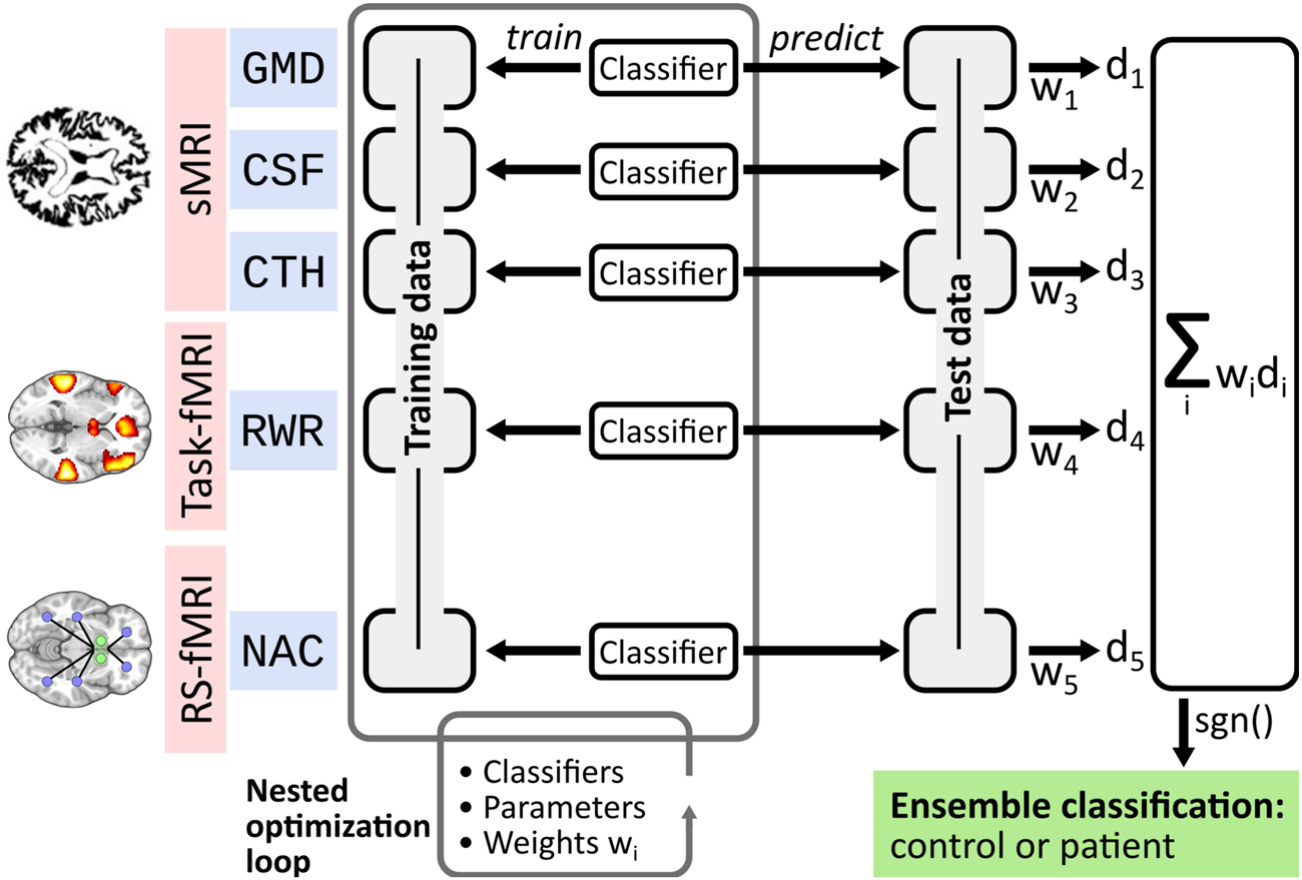
Multimodal classification scheme. Depicted is one exemplary split into training data and test data. Using a nested optimization loop, three modality-specific factors are optimized on the training data: classifier types (SVM, WeiRD), parameters (cost parameter *C* for SVM) and weights *w*_*i*_. The trained and optimized model is then applied to the test data and continuous decision values *d*_*i*_ are computed for each modality-specific classifier. The final diagnostic classification is based on a weighted sum of decision values, where weights correspond to those estimated during training.

As for unimodal classification, multimodal classification was based on soft-normalised data and proceeded in a LOO cross-validation procedure. In the multimodal scenario this meant that in each cross-validation fold, all data (i.e. all modalities) of one participant were left out for independent testing. Henceforth, we describe the analytic work-flow of training and prediction for one such fold (see also Fig. 1).

### Training

During training, a separate classifier was trained on each modality and three factors were optimized in a nested cross-validation procedure: the classifier type (SVM or WeiRD); the parameters of a classifier if applicable (cost parameter C for SVM); and modality-specific weights. In detail, for each modality separately it was first assessed whether SVM (with second-level-nested cross-validation for the optimization of C) or WeiRD were more accurate on the training data set. If SVM was superior, the optimal cost parameter was then estimated anew on the entire training data. After determining the optimal classifier for each modality, a new nested cross-validation procedure was performed for all modalities combined to estimate weighting factors for each modality. To limit computational complexity and increase robustness, each of the five modalities could be weighted only with a factor of either 1 or 2.

### Prediction

After training, the classification scheme was applied to the yet unseen test subject. Each modality-specific classifier computed a signed continuous decision value for the respective modality, where negative and positive values represented control and patient predictions and the absolute value the certainty of a classifier. The overall ensemble prediction was based on the sign of a weighted sum of modality-specific decision values with weighting factors determined during training.

To assess the effect of optimizing the classifier and the weighing scheme, the entire procedure was performed with either fixing the classifier to either WeiRD or SVM (but still optimizing C), and/or omitting modality weighting, i.e. fixing all weights to 1.

Finally, note that a key advantage of our classification scheme was that missing data were of no concern: if a subject had missing data for a modality (see Table 2 for the number of valid samples in each modality), this modality was simply omitted from the weighted sum of modality-specific decision values for the given subject.

**Table 2:** Overview of modalities.

	Time	Modality	Short	N_Ctr_	N_Pat_	No. features
sMRI	4:26	Grey-matter density	GMD	97	119	110
		Cerebrospinal fluid	CSF	97	119	11
		Cortical thickness	CTH	96	119	358
Task-based fMRI	22:10	Reward response	RWR	74	80	1461
Resting-state fMRI	6:02	Nucleus accumbens connectivity	NAC	84	93	110

Columns represent acquisition time, shortcuts for each modality used throughout the article, the number of control (N_Ctr_) and patients (N_Pat_) available for each modality, and the numbers of features per modality (No. features).

## Results

### Unimodal diagnostic classification

Three reasons motivated an initial assessment of unimodal classification, i.e. classification based on each modality individually. First, to provide a reference against which multimodal classification could be benchmarked. Second, to elucidate for each modality which brain regions contained information predictive of diagnosis. And third, to assess the agreement between different modalities with respect to patient/control predictions (inter-modality reliability).

We found that each modality discriminated significantly between patients and controls (Table 3), although the accuracy varied strongly across modalities. For both SVM and WeiRD, grey-matter density was the best-performing modality with balanced accuracies of 76.6% and 71.3%, respectively. All other modalities ranged between 55 and 66% accuracy. The balance between specificity and sensitivity was heterogenous across modalities. For instance, while cortical thickness was more sensitive than specific, cerebrospinal fluid and reward responses showed an opposite pattern.

**Table 3:** Unimodal classification.

	SVM				WeiRD			
	Acc.	Sens.	Spec.	P	Acc.	Sens.	Spec.	P
Grey-matter density	76.6	79.0	74.2	<0.001	71.3	66.4	76.3	<0.001
Cerebrospinal fluid	58.6	51.3	66.0	0.003	65.0	58.8	71.1	<0.001
Cortical thickness	54.9	58.8	51.0	0.037	65.6	69.7	61.5	<0.001
Reward response	60.2	47.5	73.0	0.002	59.3	55.0	63.5	0.005
NAcc connectivity	54.8	54.8	54.8	0.050	55.0	50.5	59.5	0.044

Classification performance of the SVM and WeiRD classifiers for the five modalities under consideration. Abbreviations: Acc. = Balanced accuracy; Sens. = sensitivity; Spec. = specificity; NAcc = Nucleus Accumbens.

**Figure 2:**
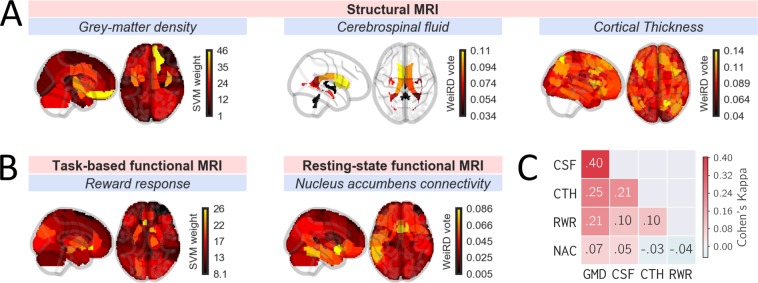
Unimodal classification. Feature importances of (**A**) structural and (**B**) functional neuroimaging modalities. Depicted are 2-*d* projections (‘glass brains’) of feature importances along the *x*- and *z*-axis. Feature importances represent SVM weights (grey-matter density, reward response) or WeiRD votes (cerebrospinal fluid, cortical thickness, resting state) depending on which classifiers was superior for a given modality. (**C**) Inter-modality reliability matrix based on Cohen’s Kappa describing the diagnostic agreement between modalities.

To investigate which brain regions contributed to classification, we inspected feature importances for each modality (Fig. 2A,B and Supplementary Fig, S1). Across modalities, we found that anterior cingulate and inferior frontal brain regions discriminated best between patients and controls. This included the cerebrospinal fluid, for which the (left) frontal ventricle was most discriminative. These results are broadly in line with the hypothesis of prefrontal cortex dysfunction in addiction^71–73^. In addition, for functional reward responses the nucleus accumbens was the second most discriminative brain region (after subcallosal anterior cingulate cortex), a key region of generic reward pathways^74^ and of bottom-up theories of addiction such as the incentive-sensitization theory^22^. Although not the focus of this study, the brain regions that discriminate best between patients and controls map well on established neural correlates of alcohol dependence^75,76^ and provide validation to the approach taken in this study.

Our initial unimodal analysis of feature importances showed that informative features were primarily located in prefrontal and cingulate brain regions (for strctural and connectivity measures) as well as in the nucleus accumbens (for reward responses).

How well do different modalities agree with respect to their diagnostic predictions? To find out, we computed inter-modality reliability scores based on Cohen’s Kappa^77^, which measures the agreement of two “raters” over and above the agreement expected by chance. For each modality we used the classifier (SVM or WeiRD) that performed better for a given modality. As shown in Fig. 2C, the agreement was generally highest between modalities based on structural MRI. Within those, predictions based on grey-matter density and cerebrospinal fluid showed the highest agreement with a Kappa value of 0.4 (considered a ‘moderate‘ agreement^78^). All other modality comparisons exhibited relatively little agreement with Kappa values <= 0.25. Across all modalities, predictions based on nucleus accumbens connectivity showed the least agreement with any other modality.

### Multimodal diagnostic classification performs better than each individual modality

To combine modalities for multimodal classification, we trained individual classifiers on each modality separately, computed predictions based on each modality, and then combined these predictions in the form of a weighted ensemble vote (Fig. 1). A number of details of this analytic framework deserve highlighting. First, classifiers were optimized for each modality individually, both with respect to the classifier type (SVM or WeiRD) and in terms of a modality-specific regularization parameter (C in the case of SVM). Second, instead of relying on discrete predictions, continuous decision values were computed for each modality, which enabled a more fine-grained ensemble decision. Third, on top of these intrinsic classifier-based decision values, an external weighting matrix across modalities was learned from the training data, further refining the ensemble vote.

Employing this fully-featured multimodal classification scheme yielded a balanced accuracy of 79.3% (p < 10^−18^). Thus, combining the information from multiple modalities improved the accuracy compared to the best individual modality (grey-matter density: 76.6%). An analysis of sensitivity and specificity showed that this improvement was due to an increase of specificity (81.3%; sensitivity: 77.3%), which reached a level unmatched by any individual modality.

**Figure 3:**
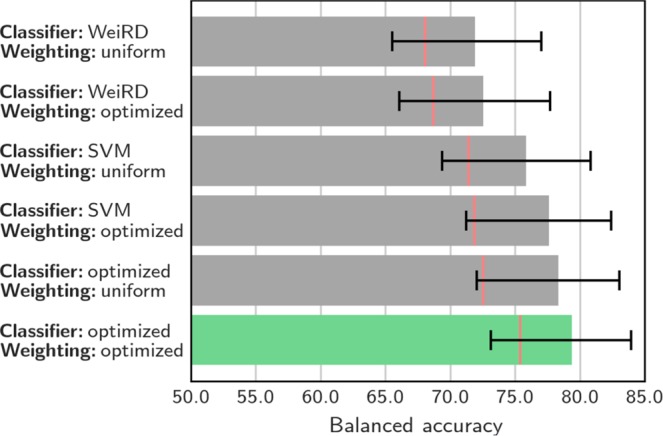
Multimodal classification. Balanced accuracy for classification schemes based on different classifier configurations (SVM, WeiRD or optimized between SVM and WeiRD) and with uniform (i.e., all weights set to 1) or optimized weighting of modalities. Optimizing both classifiers and weighting yielded the best performance (highlighted in green). Red lines indicate the balanced accuracy when ensemble prediction was based on discrete “control” and “patient” judgements instead of continuous decision values. Error bars represent the 95% posterior probability interval^67^.

To investigate which analytic component of the multimodal classification scheme was essential for this improvement – or which aspect may have been redundant – we computed accuracies while muting one or more of the features. As shown in Fig. 3, we found that indeed each analytic component discussed above (optimization of classifiers, decision values instead of discrete predictions, cross-modal weighting) was responsible for an incremental improvement of performance. Of these, the largest improvement was due to considering continuous decision values instead of binary “control” and “patient” predictions (represented by red lines in Fig. 3): without this feature, the multimodal accuracy would have dropped below the best individual modality, even if classifiers and weights were optimized (75.4%). Overall, these results show that multimodal classification requires a nuanced integration of modalities in order to achieve a meaningful benefit.

To assess the significance of each individual modality for multimodal classification, we computed classification accuracies while tentatively excluding each modality once. The results (Table 4) showed that the accuracy dropped in each case, indicating that each modality was important for overall multimodal performance. Not surprisingly, the sharpest drop in performance was observed when excluding the best individual modality, grey-matter density (−9.6%). In particular, grey-matter density was largely responsible for the high sensitivity of multimodal classification, evidenced by a substantial drop of sensitivity when excluded (−18.1%). By contrast, the sharpest drop in specificity was found when excluding cerebrospinal fluid (−7.3%). Taken together, these results show that the success of multimodal classification was based on the combined information of all modalities such that no single modality was effectively redundant.

**Table 4:** Impact of excluding modalities.

Acc. (Δ)	Sens. (Δ)	Spec. (Δ)
69.7 (−9.6)	59.2 (−18.1)	80.2 (−1.0)
75.3 (−4.0)	76.7 (−0.6)	74.0 (−7.3)
78.1 (−1.2)	75.8 (−1.5)	80.4 (−0.8)
77.0 (−2.3)	75.6 (−1.7)	78.4 (−2.9)
77.3 (−2.0)	77.3 (0.0)	77.3 (−3.9)

Performance of multimodal classification when leaving out each modality once (Δ = change with respect to the full model). Abbreviations: Acc. = Balanced accuracy; Sens. = sensitivity; Spec. = specificity; NAcc = Nucleus Accumbens.

## Discussion

In the present investigation we used structural, functional task-based and functional resting-state MRI to construct a diagnostic machine-learning classifier for alcohol dependence. A novel multimodal classification scheme, in which modality-specific weightings and optimal classifiers were estimated from training data, slightly outperformed the strongest individual modality and achieved a balanced accuracy of 79.3%.

Our initial unimodal analysis of feature importances showed that informative features were primarily located in (orbito-)prefrontal and cingulate brain regions as well as in the nucleus accumbens (for basic reward responses). These foci are broadly in line with previous investigations into brain structure and function in alcohol dependence, which identified executive and reward networks as major neural circuits that are affected in patients^75^. Thus, our unimodal structural and functional data show diagnostic characteristics well in line with previous results. Despite the overlap of affected brain structures, none of these considered modalities was redundant: multimodal classification accuracy was reduced for each modality that was tentatively excluded. Together, these exploratory analyses into individual modalities thus attest to 1) plausible unimodal between-group effects and 2) a sensible selection of modalities that leveraged on non-redundant sources of information.

In comparison to the best individual modality (grey-matter density with SVM: 76.6%), multimodal classification yielded an improvement of 2.7% accuracy, corresponding to a net gain of 6 additional subjects that were correctly classified. This gain in accuracy was specifically due to an increase in specificity (81.3%), which was 7% higher than for grey-matter density (74.2%). The specific increase of specificity is noteworthy, as we recently found a particular advantage of unimodal computer-based classification over human judgements with respect to *sensitivity*, while *specificity* was higher for the judgements of an experienced radiologist (81.4%^16^). Thus, when combining multiple modalities, computer-based classification matched human performance in terms of identifying true negatives, thereby eliminating a weakness of unimodal classification.

Although the improvement in overall accuracy is modest at 2.7%, one important qualification is that the comparison to the best of several modalities is necessarily unfair due to selection bias; that is, it is likely that the accuracy of the best modality is – to some degree – inflated by noise. Besides this ‘double dipping’^79^ bias, even at 2.7% our observed benefit of multimodal classification is noteworthy, as many previous studies have failed to find any advantage of combining multiple modalities^9,10,80,81^. There is currently no consensus on *why* an effective combination of multiple MRI modalities appears to be a surprisingly intricate task. But clearly, the success of multimodal classification must depend on how individual modalities complement each other: if individual modalities perfectly agree with each other, they are effectively redundant and thus no multimodal benefit is expected; vice versa, if predictions of individual modalities are completely uncorrelated, there is no positive cumulative effect through the combination of modalities. In the present case, the agreement of predictions between different modalities was quite variable overall but tended towards low inter-modality reliabilities (Cohen’s Kappa scores up to 0.4), which reflects the moderate benefit of multimodal classification.

At a methodological level, we found a number of analytic choices to be critical for the integration of multiple modalities. First, as data from different modalities will naturally have different structural properties, it is likely that there is no single classifier type that fits all modalities. Yet, to our knowledge, while multimodal investigations often *compare* classifier types^9,11,82^, they do so by applying the same classifier to all modalities, i.e. they do not optimize the classifier type in a modality-specific manner. Here we found that optimizing the classifier type for each modality was superior to using either SVM or WeiRD uniformly across modalities. Second, applying a weight matrix learned from training data to the ensemble prediction likewise increased the performance compared to unweighted integration. Although this particular aspect has been considered previously (e.g. by means of logistic regression on predictions of individual modalities^11^), our results corroborate the importance of this analytic step.

Third, the biggest gain in accuracy was owed to using continuous classifier decision values instead of discrete (binary) predictions. The benefit of decision values can be explained by the fact that for both SVM and WeiRD, decision values reflected the certainty of the classifier. Thus, more certain predictions factor in more strongly into the ensemble prediction and thereby improve the overall accuracy. Indeed, we have previously shown that valuable information is contained in unthresholded decision values of classifiers applied to neuroimaging data^63^. On the basis of our results we thus recommend to avoid discretizing modality-specific predictions and instead to utilize fine-grained information contained in classifier decision values.

Despite these methodological insights about combining multiple neuroimaging modalities from both structural and functional MRI, considering the fact that fMRI scanning was more time-consuming and involved more elaborate data analysis, the achieved improvement through fMRI must be debated in view of clinical practicability and a cost-benefit analysis. In our view, several factors are important for fMRI to become part of a clinically realistic diagnostic imaging battery. First, an accuracy net gain in the order of a few percent is relatively small, given the additional effort of instructing and conducting a functional scan in addition to a structural scan. Thus, to justify task-based and resting-state fMRI measures, these must either be more sensitive – or more orthogonal to the information from structural MRI. Second, employed fMRI tasks have to be sufficiently brief. This would be a relatively simple optimization of the present imaging battery, as the basic reward signal used for classification was extracted from a relatively complex decision-making task which could be condensed substantially. Third, at present the analysis of functional MRI data is more laborious compared to the analysis of structural MRI data. However, with the development of standardized, efficient, and robust analysis protocols (e.g. fMRIprep^83^), fMRI could become a realistic option for day-to-day clinical diagnosis. In sum, in terms of direct clinical applicability, currently the most realistic neuroimaging-based classifier for AD may be unimodal based on structural MRI and grey-matter density specifically.

A number of limitations should be noted. First, our sample is predominantly male, which limits the generalization of our results to female patients. This may be especially relevant for the most predictive modality in our approach, grey-matter density, as numerous studies have shown greater sensitivity to the neurotoxic effects of alcohol on grey matter in women^84–86^. Second, we were not able to validate our results against an independent sample, as the multimodal imaging battery employed by the LeAD study has no precedence in the AD literature. However, in previous work on unimodal imaging^16^ we found almost perfect generalization to an independent sample (original: 74%; generalization: 73%). Although these previous results cannot be directly extrapolated to our current work, we note that both investigations share major aspects of the methodology, including the use of the weighted robust distance classifier^62^ and cross-validation with nested cross-validation for tuning parameters.

Third, the selection of neuroimaging modalities is not exhaustive. While in the present work we focused on a parsimonious set of established but basic modalities, especially in the functional domain there is abundant literature on various functional correlates of causes, state markers (e.g. craving) and consequences in alcohol dependence^75,87^. For instance, the influential iRISA (impaired response inhibition and salience attribution) model proposes that disrupted function of the prefrontal cortex leads not only to attributing excessive salience to drugs and associated cues, but also impairs the ability to inhibit drug-related behaviours^71^. More complete neuroimaging models of alcohol dependence could thus additionally consider a prefrontal functional correlate of cognitive control. Regarding functional connectivity, there is evidence that large-scale functional networks (e.g. default mode network or cognitive control network) explain a substantial amount of variance with respect to alcohol use severity and may as well be informative for diagnostic classification^88^. These large-scale networks were not considered in the present study and thus deserve further research in the context of diagnostic neuroimaging-based classification. Fourth, based on the cross-sectional design in this study one cannot infer whether the neurobiological differences utilized by our classifier are causes, state markers or consequences of alcohol dependence (although see preliminary evidence for the latter possibility in Supplementary Fig. 2). Thus, our classifier provides no mechanistic insight into the pathogenesis of alcohol dependence. For instance, based on previous findings it is possible to that group differences in brain structure may be predominantly consequences of severe alcohol abuse, as these changes partially reverse during abstinence^89–91^; differences in brain function may, in turn, predispose for addiction or relapse from addiction^30,92^. From a strict machine-learning point of view, one may be tempted to treat the issue of mechanistic insight as secondary. However, although neural features that characterize a predisposition are particularly valuable due to their prognostic potential, for diagnosis they bear the risk of misclassifying healthy individuals that did not develop AD despite having a disadvantageous predisposition. Patients which developed AD without such a predisposition could likewise be misclassified due to these features. For this reason, the ‘chicken or egg’ causality dilemma is indeed relevant for machine-learning-based diagnosis and should be investigated in future longitudinal studies.

Finally, in view of modern frameworks of psychiatric disease such as the Research Domain Criteria (RDoC) project, in which psychiatric phenotypes are defined as “spanning the range from normal to abnormal”^93,94^, the present approach could be readily adapted to predict dimensional markers of disease. Analogous to the idea of a weighted voting scheme across classifiers applied here for the case of binary prediction, multiple modality-specific regression models would be trained and their outputs combined to form a continuous ensemble prediction. The ensemble prediction would likewise be based on a weighted (and normalized) sum of individual predictions. Conceivable dimensional markers for the case of alcohol dependence are the magnitude of craving in acute addiction^95^, biological markers such as serum levels of carbohydrate deficient transferrin (CDT) and gamma glutamyltransferase (GGT)^96^, or scores of clinical questionnaires such as the Alcohol Use Disorders Identification Test (AUDIT^97^). Overall, we conclude that the combination of multiple neuroimaging modalities is able to moderately improve the accuracy of machine-learning-based diagnostic classification in alcohol dependence. Our results allow us to make several methodological recommendations for the exploitation and integration of different modalities with the goal to compute optimal ensemble predictions, thereby paving the way towards more effective multimodal neuroimaging classifiers. Yet, at present, given the strong predictive performance of grey-matter density alone and taking a cost-benefit analyses into account, we currently recommend to focus on structural MRI for the diagnostic classification of alcohol dependence.

## Supplementary information

Supplementary Information.

## Data Availability

Code and data used in the current study are available from the corresponding author on reasonable request.
