# Supplementary material for: A multimodal neuroimaging classifier for alcohol dependence
Source: Sci Rep. 2020 Jan 15;10:298. doi: 10.1038/s41598-019-56923-9 (PMC6962344; doi:10.1038/s41598-019-56923-9)
Supplement: Supplementary file 1 — Supplementary Information. [file 41598_2019_56923_MOESM1_ESM.docx]

**Supplementary material to: A multimodal neuroimaging classifier for alcohol dependence**

Matthias Guggenmos^1^, Katharina Schmack^1^, Ilya M. Veer^1^, Tristram Lett^1^, Maria Sekutowicz^1^, Miriam Sebold^1^, Maria Garbusow^1^, Christian Sommer^2^, Hans-Ulrich Wittchen^4,5^, Ulrich S. Zimmermann^2^, Michael N Smolka^2,3^, Henrik Walter^1^, Andreas Heinz^1^, Philipp Sterzer^1^

^1^ Department of Psychiatry and Psychotherapy, Charité Universitätsmedizin, Germany

^2^ Department of Psychiatry and Psychotherapy, Technische Universität Dresden, Germany

^3^ Neuroimaging Center, Technische Universität Dresden, Dresden, Germany

^4^ Institute of Clinical Psychology and Psychotherapy, Technische Universität Dresden, Germany

^5^ Department of Psychiatry and Psychotherapy, Ludwig Maximilans Universität Munich, Munich, Germany


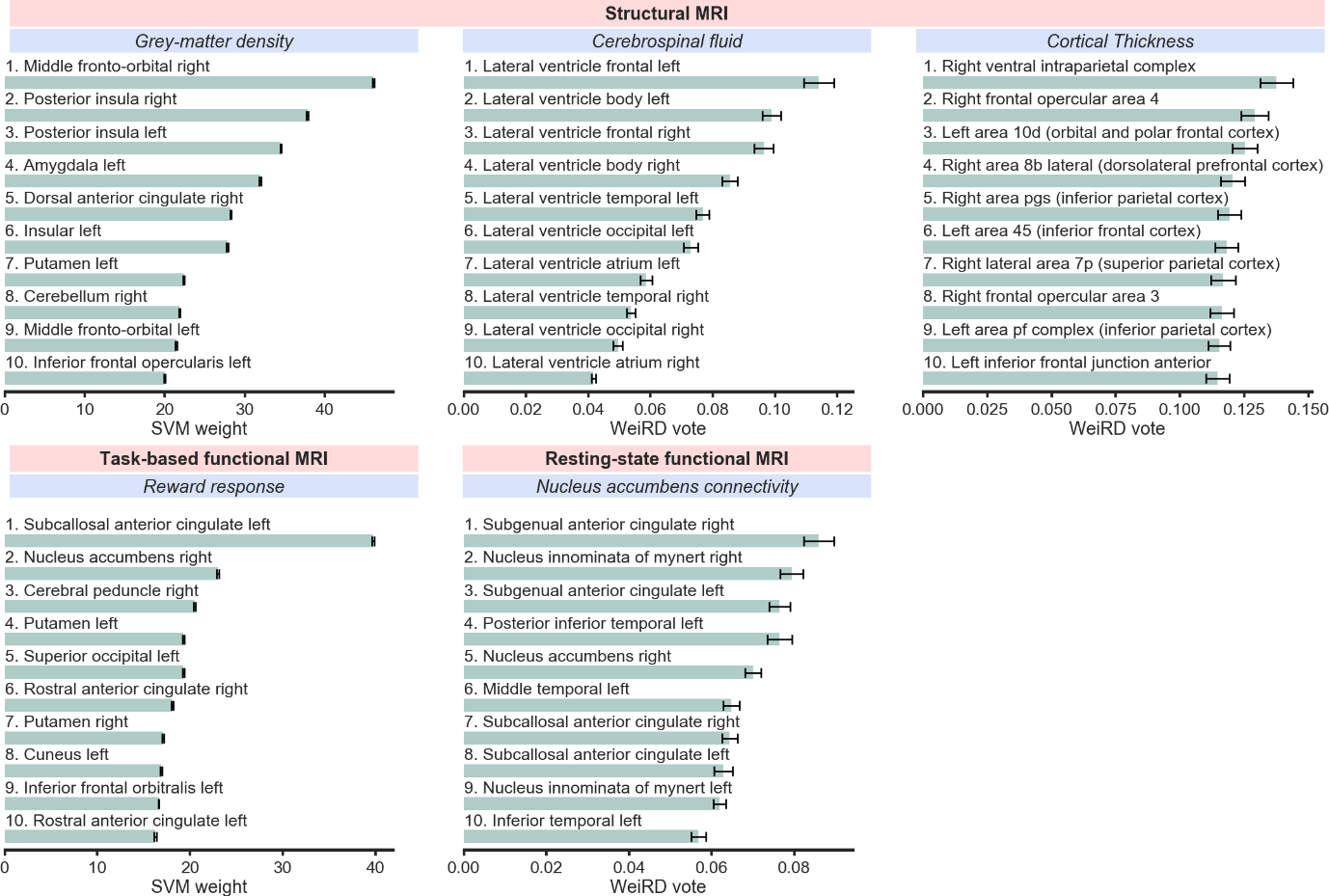


**Supplementary Figure S1. Feature importances.** Depicted are for each modality the 10 most important brain regions for unimodal classification. Feature importance is characterized by SVM weights in case of SVM classification (grey-matter density, reward responses) and by WeiRD votes in case of WeiRD classification (cerebrospinal fluid, cortical thickness, resting state connectivity). Error bars represent the 95% confidence interval across cross-validation folds.


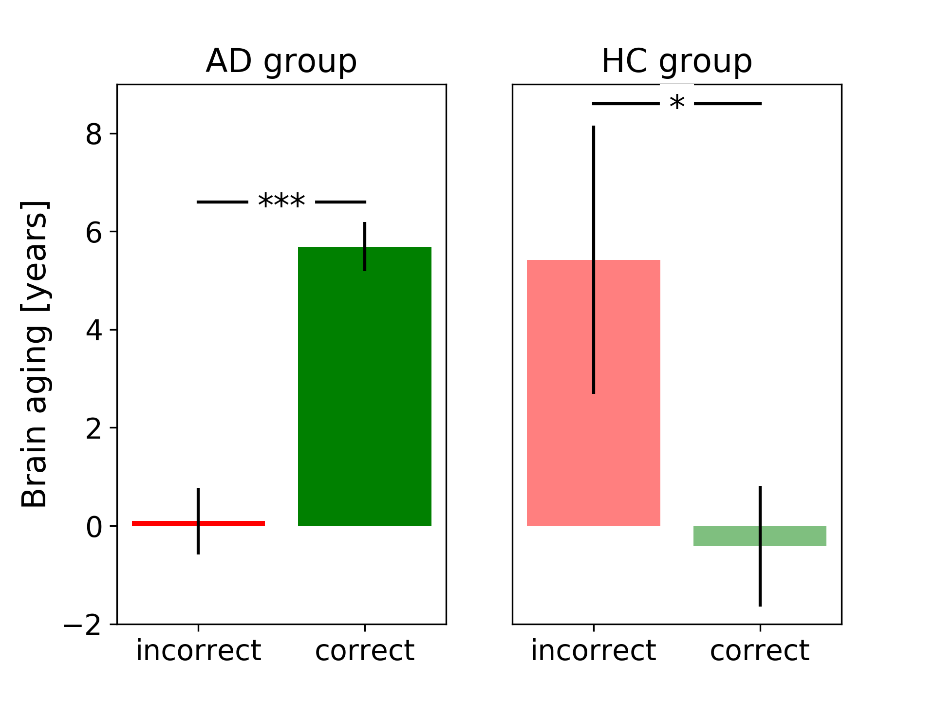


**Supplementary Figure S2. Relationship between brain aging and classifier predictions.** Brain aging quantifies the difference between chronological age and biological age estimated on the basis of grey-matter volume. Brain aging estimates are derived from a previous study based on the same sample [Guggenmos et al., 2017. Quantitative neurobiological evidence for accelerated brain aging in alcohol dependence. Transl. Psychiatry 7. doi: 10.1038/s41398-017-0037-y]. Brain aging in correctly predicted patients (5.7 ± 0.5 years) was significantly more pronounced than in patients incorrectly predicted as controls (0.1 ± 0.7 years) (paired t-test: t_117_ = −5.6, p < 0.001). In controls, this pattern of results was reversed: brain aging was higher in incorrectly (5.4 ± 2.7 years) compared to incorrectly (−0.42 ± 1.2 years) classified controls (t_94_ = 2.0, p=0.045). This provides suggestive evidence that predictions of the multimodal classifier were largely based on neurotoxic consequences of alcohol dependence.
